# Dissemination and characteristics of carbapenem-resistant *Klebsiella pneumoniae* in nine district hospitals in southwestern China

**DOI:** 10.3389/fmicb.2023.1269408

**Published:** 2023-10-24

**Authors:** Yonghong Wang, Yan Ouyang, Xiuyu Xu, Shan Sun, Xiaolang Tian, Hang Liu, Yun Xia

**Affiliations:** ^1^Department of Clinical Laboratory, Chongqing Qianjiang Central Hospital, Chongqing University Qianjiang Hospital, Chongqing, China; ^2^Qianjiang Key Laboratory of Chongqing Qianjiang Central Hospital Laboratory Medicine, Chongqing, China; ^3^Department of Laboratory Medicine, The First Affiliated Hospital of Chongqing Medical University, Chongqing, China; ^4^Department of Nursing, Union Hospital, Tongji Medical College, Huazhong University of Science and Technology, Wuhan, China; ^5^Department of Clinical Laboratory, The Fifth People’s Hospital of Chongqing, Chongqing, China; ^6^Department of Clinical Laboratory, Institute of Translational Medicine, Renmin Hospital of Wuhan University, Wuhan, China

**Keywords:** CRKP, district hospitals, dissemination and characteristics, HV-CRKP, resistance mechanisms

## Abstract

**Background:**

Carbapenem-resistant *Klebsiella pneumoniae* (CRKP) is epidemically transmitted globally, but few studies focused on the prevalence in district-level hospitals. In this study, we investigated CRKP strains collected from nine district hospitals from September 2019 to September 2020, aiming to determine the resistance mechanisms, virulence profiles, and molecular epidemiological characteristics of CRKP in district hospitals in Southwest China.

**Methods:**

A total of 51 CRKP strains were collected from 9 district-level hospitals. Matrix-assisted laser desorption/ionization-time of flight mass spectrometer was used for strain identification review, and the micro-broth dilution method was used for antibiotic sensitivity detection. Molecular epidemiological investigation of strains was performed by multilocus sequence typing (MLST) and pulsed-field gel electrophoresis (PFGE) methods. PCR and efflux pump inhibition tests were used to detect CRKP resistance mechanisms. PCR and serum killing tests were used to detect capsular serotype, virulence-related genes, and virulence validation.

**Results:**

The CRKP strains in district hospitals presented high levels of MIC_50_ and MIC_90_ in carbapenem antibiotics especially ertapenem and meropenem. A total of 90.2% (46/51) CRKP strains were detected as carbapenemase producers, and the proportion of strains co-expressing carbapenemases was 11.8% (6/51). All CRKP strains were grouped into eight MLST types, and ST11 was the most prevalent genotype. A total of 11.8% (6/51) CRKP isolates were positive for the string test, and three strains of hypervirulent and carbapenem-resistant *K. pneumoniae* (HV-CRKP) were positive in serum killing test. The molecular typing of all the CRKP isolates was grouped into 29 different PFGE patterns, and 40 ST11 isolates belonged to 20 different PFGE clusters.

**Conclusion:**

CRKP strains showed high-level antibiotic resistance and virulence phenotype in district hospitals in Southwest China, which suggested that we should immediately pay attention to the rapid dissemination of the CRKP in regional hospitals. Our study will provide new insights into the epidemiology of CRKP in regional hospitals, which will help regional hospitals develop nosocomial infection prevention and control policies tailored to local conditions.

## Highlights

– Current studies on the epidemiology and antimicrobial-resistant characteristics of CRKP and/or HV-CRKP mainly concentrated on large tertiary teaching hospitals and ignored the district-level hospitals.
– In our study, we found that CRKP strains exhibited a high level of antibiotic resistance, a high proportion of harboring carbapenemases, and hypervirulence phenotype in district hospitals in Southwest, China.
– These results will further provide support for the prevention and control of nosocomial infection by CRKP in district-level medical institutions.

## Introduction

*Klebsiella pneumoniae* is one of the most common and conditionally pathogenic microorganisms causing healthcare-associated infections. The rapid prevalence and outbreak of the Carbapenem-resistant *K. pneumoniae* (CRKP) strain severely threatens public health and become a great global concern (Zong et al., 2020; Luterbach et al., 2023). The CRKP isolates dominate all hospital-acquired Carbapenem-resistant *Enterobacteriaceae* (CRE) strains in Europe and China, up to 85 and 73.9%, respectively (Logan and Weinstein, 2017; Zhang et al., 2018). Moreover, CRKP nosocomial infections dramatically increase the in-hospital mortality rate and medical expenditure (Zhen et al., 2020; Zhang et al., 2021). The mechanisms of resistance to carbapenem mainly included the production of carbapenemases, upregulation of *ESBLs*, overexpression of *AmpC* gene combined with a deficiency of outer membrane porins, and overproduction of efflux system (Nordmann and Poirel, 2019; Lan et al., 2021). However, the epidemiological characteristics and resistant mechanisms of CRKP strains were not exactly identical geographically and highly associated with patients’ clinical outcomes (Hu et al., 2020; Wang et al., 2022; Wyres and Holt, 2022), suggesting that findings from one region may not be directly generalizable to other regions.

Currently, research on the epidemiology and antimicrobial-resistant characteristics of CRKP and/or hypervirulent and carbapenem-resistant *K. pneumoniae* (HV-CRKP) primarily focused on large tertiary teaching hospitals, and less attention is paid to district-level hospitals (Hu et al., 2020; Lan et al., 2021). A global survey presented that 98% of Chinese CRKP strains and 88% of American CRKP strains carried at least one type of carbapenemases, conferring *K. pneumoniae* resistance to carbapenem antibiotics (Wang et al., 2022), but the hospital levels of the sample source were not distinguished. The majority of the carbapenemase genes separated from CRKP isolates around the world were *bla*_KPC_ genes, but their positive ratios varied greatly ranging from 4 to 94%, while *bla*_KPC_ gene-mediated resistance exhibited an overwhelming tendency in Chinese tertiary teaching hospitals (Hu et al., 2020; Zeng et al., 2021; Wang et al., 2022). Regarding the epidemiology of clinical CRKP strains, the ST258 genotype is most widely distributed in the United States and Israel (Wyres and Holt, 2022). In comparison, the ST11 genotype was identified as the dominant clone in China, accounting for approximately 60% of CRKP strains (Yao et al., 2015; Zhan et al., 2017). Distinct from classical CRKP, the emerging hypervirulent *K. pneumoniae* (HVKP) strains, well confirmed as a clinically causative agent for pyogenic liver abscesses, have been reported worldwide in the last decade. More gravely, the occurrence and eruption of a mortal ST11 genotype HV-CRKP have been detected in several Chinese clinical settings (Gu et al., 2018; Yang et al., 2022). A study of CRE prevalence in secondary hospitals and children’s hospitals in Nanjing, China presented that CRE strains in regional hospitals exhibited multiple resistance determinants and plasmid replicons (Zhou et al., 2020), but the virulence profile has not been investigated.

Due to the lack of standardized use of antibiotics, high-level medical conditions, and high-quality nosocomial infection prevention and control, district-level hospitals were always facing the crisis of CRKP outbreaks. If these superbug-resistant bacteria were ignored, they would eventually spread across regional hospitals, and seriously endanger the patients’ lives and health conditions. Therefore, the objective of this study was to investigate the molecular epidemiological feature, resistance mechanisms, and virulence status of CRKP in nine district hospitals in Chongqing municipality. These findings will provide an important basis for formulating effective measures to suppress the rapid spread of CRKP and HV-CRKP strains in district hospitals in China and other developing countries and regions’ medical institutions.

## Materials and methods

### Strains collection and identification

From September 2019 to September 2020, a total of 51 non-duplicate CRKP isolates were isolated from various specimens of patients successively in 9 distinct hospitals in Chongqing, China. The CRKP strains were sourced from the following distinct hospitals: Wanzhou Three Gorges Central Hospital (*n* = 16), Dianjiang People’s Hospital (*n* = 9), Youyang County People’s Hospital (*n* = 4), Qianjiang Central Hospital (*n* = 4), Fengdu People’s Hospital (*n* = 5), Jiangjin Central Hospital (*n* = 4), Chongqing Ninth People’s Hospital (*n* = 3), Qijiang District People’s Hospital (*n* = 3), and People’s Hospital of Banan District (*n* = 3). We categorized the samples into three distinct groups based on the timeline of CRKP acquisition: hospital acquired (HA), community acquired (CA), and healthcare associated (HCA). HA-CRKP refers to isolates obtained from patients who have been hospitalized for more than 48 h and did not exhibit any signs or symptoms of infection upon admission; CA-CRKP pertains to isolates obtained from patients within 48 h of admission who did not exhibit any signs or symptoms of infection during the 3 months prior to admission and had no recent contact with healthcare systems; HCA-CRKP encompasses isolates obtained from patients within 48 h of admission who had recent healthcare system contact within the preceding 3 months, received regular hemodialysis, recently underwent intravenous antibiotic therapy or chemotherapy, or were hospitalized in an acute care facility for more than 2 days in the 3 months prior to CRKP isolation (Lau et al., 2021). All isolates were identified at the species level and routine antimicrobial susceptibility testing was performed by using the VITEK2 compact or VITEK MS (bioMerieux, Hazelwood, MO, United States) automated system. The isolates were collected by the rapid freezing method and stored at −80°C for further analysis. Isolates were included in this study if they were resistant to at least one of the carbapenems by the broth microdilution method, with the criteria of minimal inhibitory concentrations (MICs) of ≥2 μg/ml for ertapenem, ≥4 μg/ml for imipenem, or ≥4 μg/ml for meropenem.

### Antimicrobial susceptibility testing

All isolates underwent antibiotic susceptibility testing, where we determined the MICs using the broth microdilution method. The antibiotics tested included: ertapenem (ETP), imipenem (IPM), meropenem (MEM), colistin (CST), tigecycline (TGC), aztreonam, gentamicin, amikacin, ciprofloxacin, ceftazidime, cefepime, and ceftazidime/avibactam. The majority of antibiotic breakpoints used for interpretation were recommended by the CLSI (2021). The interpretive criterion for tigecycline was based on the identified interpretive criteria of the Food and Drug Administration, with the interval MIC of ≤2 μg/ml and ≥8 μg/ml considered as the susceptibility and resistance breakpoints. Quality control was managed by using *Escherichia coli* ATCC 25922. MIC_50_, MIC_90_, and the MIC range of each tested agents were also analyzed in our study.

### Detection of carbapenem resistance genes

PCR was performed to detect the presence of carbapenemase-related genes, including *bla*_KPC_, *bla*_NDM_, *bla*_VIM_, *bla*_IMP_, and *bla*_OXA–48_. In addition, *ESBLs*, *AmpC*, *aminoglycoside*, and fluoroquinolone resistance genes, and *ompK35* and *ompK36* genes were also identified. The primers were as described in our previous studies and listed in Supplementary Table 1 (Liu et al., 2019; Zou et al., 2020), and all positive PCR products were used for Sanger sequencing to confirm these gene sequences and variants.

### Phenotypic detection of carbapenemase and efflux pump inhibitory assay

The carbapenemases phenotype was determined by the modified carbapenem inactivation method (mCIM) test recommended by the CLSI (2021). To assess the role of efflux pumps in non-carbapenemase-producing-CRKP isolates, the efflux pump inhibitors (EPI): carbonyl cyanide m-chlorophenylhydrazone (CCCP, 16 μg/ml, Sigma) and Phe-Arg-β-naphthylamide (PAβN, 20 μg/ml, Sigma), were selected to investigate efflux function of strains to carbapenem antibiotics by using the standard broth microdilution method. Compare with the absence of EPI, the MIC value of any antibiotic in ETP, IPM, and MEM was reduced by at least four times after the addition of EPI, which was considered to be a significant inhibition of the efflux pumps (Liu et al., 2019).

### Hypermucoviscosity phenotype detection and serum killing assay

The carbapenem-resistant isolates were subcultured overnight on blood agar at 37°C. Isolates were considered positive for the hypermucoviscosity phenotype if an inoculation loop touched to the surface of the colony generated a viscous string of 5 mm in length when pulled away from the colony (Lee et al., 2006). CRKP strains with a positive string test were designated HV-CRKP. Serum killing assay was conducted to determine the virulence *in vitro* as previously described (Soto et al., 2016). An inoculum of 25 μl prepared from the mid-log phase was diluted by 0.9% saline solution and was added to 75 μl of pooled human sera contained in a 10 × 75 mm Falcon polypropylene tube. Viable counts were checked at 0, 1, 2, and 3 h of incubation at 37°C. The mean results were expressed as percentage of inoculation and a strain was classified as serum sensitive, intermediately sensitive, and resistant. The *K. pneumoniae* ATCC700603 was used as a standard control strain. One previously confirmed non-hypervirulent CRKP-1 strain in our laboratory was selected as negative control strains for the serum killing assay.

### Capsular serotyping and detection of virulence factors

Capsular serotypes (K1, K2, K5, K20, K54, and K57) of these HV-CRKP strains were detected as previously described and primers were listed in Supplementary Table 1 (Ssekatawa et al., 2021). Additionally, eighteen virulence-associated genes including *iutA*, *entB*, *irp-1*, *irp-2*, *fyuA*, *ybtS*, *fimH*, *iroN*, *kpn*, *mrkD*, *ycfM*, *rmpA*, *magA*, *aerobactin*, *traT*, *wcaG*, *cnf-1*, and *hlyA* were detected by PCR and DNA sequencing among these HV-CRKP isolates (Tang et al., 2020).

### Molecular epidemiological study

Pulsed-field gel electrophoresis (PFGE) was performed as previously described in all the CRKP strains, and banding patterns were interpreted according to the recommended criteria (Tenover et al., 1995; Liu et al., 2019). The DNA sequences of seven housekeeping genes including *gapA*, *infB*, *mdh*, *pgi*, *phoE*, *rpoB*, and *tonB* for *K. pneumoniae* were amplified and sequenced for multilocus sequence typing (MLST) alignment (Tang et al., 2020). Sequence types (STs) were identified by the online database on the Pasteur Institute MLST website.^1^ The MLST primers were presented in Supplementary Table 1.

### Statistical analysis

All analyses were performed using SPSS v.20.0 software (SPSS Inc., Chicago, IL, USA). Categorical variables, expressed as numbers and percentages, were compared by the Chi-square or Fisher’s exact test. A value of *P* < 0.05 was considered statistically significant.

## Results

### General characteristics and antimicrobial susceptibility of CRKP isolates

A total of 51 strains were identified that were resistant to at least one of the carbapenems and met the study criteria for CRKP. These non-duplicated isolates were mainly cultured from sputum (*n* = 27), urine (*n* = 15), blood (*n* = 7), and wound secretion (*n* = 2). In addition, 78.4% (40/51) of CRKP strains were hospital-acquired, while smaller proportions included community-acquired infections (5.9%, *n* = 3) and healthcare-associated infections (15.7%, *n* = 8). As shown in Table 1, all CRKP isolates were observed in ertapenem resistance, while 94.1% of the strains presented resistance to meropenem and imipenem. Carbapenemase-positive strains accounted for 90.2% of all CRKP strains (46/51). Compared with carbapenemase-negative isolates, carbapenemase-positive CRKP isolates exhibited higher proportions and levels resistance to imipenem and meropenem. In carbapenemase-producing CRKP strains, the MIC_50_ of the three carbapenem antimicrobials ertapenem, imipenem, and meropenem were 256, 64, and 128 μg/ml, respectively. Fortunately, they showed high susceptibility to colistin and tigecycline, with 88.2 and 96.1%, respectively. Interestingly, some carbapenemase-negative CRKP isolates showed high sensitivity to imipenem (3/5, 60%) and meropenem (3/5, 60%). The MIC_50_ and MIC_90_ of imipenem and meropenem were much lower than carbapenemase-positive strains, and fully sensitive to colistin (5/5, 100%) and tigecycline (5/5, 100%). Moreover, the infants and elderly were more prone to CRKP infection. It is noteworthy that 37.3% (19/51) of CRKP infection patients have died or given up treatment, which indicates high mortality and poor prognosis after CRKP infection in district hospitals (Supplementary Table 2).

**TABLE 1:** Antimicrobial susceptibility of CRKP isolates with or without carbapenemase.

Antimicrobial agents	Total (*N* = 51)	Carbapenemase positive (*N* = 46)				Carbapenemase negative (*N* = 5)				*P*-value
	*R* (%)	*R* (%)	MIC_50_	MIC_90_	Range	*R* (%)	MIC_50_	MIC_90_	Range	
Ertapenem	51 (100)	46 (100)	256	512	8–512	5 (100)	4	64	4–64	–
Imipenem	48 (94.1)	46 (100)	64	128	4–512	2 (40.0)	0.5	32	0.5–32	**<0.001**
Meropenem	48 (94.1)	46 (100)	128	256	4–256	2 (40.0)	0.5	16	0.5–16	**<0.001**
Cefepime	50 (98.0)	46 (100)	128	512	64–512	4 (80.0)	64	512	8–512	0.098
Ceftazidime	51 (100)	46 (100)	256	512	64–512	5 (100)	256	512	64–512	–
Amikacin	37 (72.5)	33 (71.7)	64	256	4–512	4 (80.0)	64	256	8–256	1.0
Gentamicin	42 (82.4)	38 (82.6)	32	128	1–256	4 (80.0)	32	128	2–128	1.0
Aztreonam	51 (100)	46 (100)	256	512	32–512	5 (100)	128	512	32–512	–
Ciprofloxacin	46 (90.2)	41 (89.1)	8	32	0.125–32	5 (100)	8	32	2–32	1.0
Ceftazidime/avibactam (AVI 4)	17 (33.3)	17 (37.0)	2	128	0.125–128	0 (0.0)	0.5	4	0.125–4	0.156
Colistin	6 (11.8)	6 (13.0)	1	4	1–4	0 (0.0)	1	2	1–2	1.0
Tigecycline	2 (3.9)	2 (4.3)	1	4	0.5–16	0 (0.0)	2	4	2–4	1.0

Data are number resistant (% of resistance rates). *P*-value for comparisons of the resistance rates of carbapenemase-positive and carbapenemase-negative groups. Bold face indicates values that are significant (*P* < 0.05). *R*, resistance. Ceftazidime/avibactam (AVI 4): avibactam was tested at a fixed concentration of 4 mg/L in combination with doubling dilutions of ceftazidime.

### Molecular analysis of carbapenem resistance mechanisms

**FIGURE 1:**
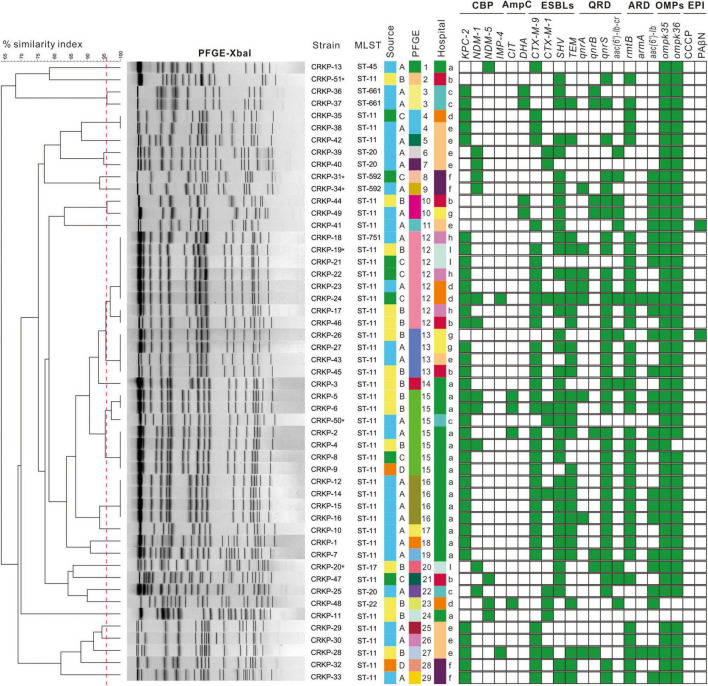
Dendrogram of pulse-field gel electrophoresis developed using BioNumerics software for 51 CRKP isolates. Clusters were defined as DNA patterns sharing ≥96.6% similarity. Strain numbers, MLST, source of initial isolation, hospital information, and resistance determinants are included along each PFGE lane. MLST, multilocus sequence typing; CBP, carbapenemase; ESBLs, extended spectrum beta-lactamases; QRD, fluoroquinolone resistant determinants; ARD, aminoglycoside resistant determinants; OMPs, outer membrane proteins; EPI, efflux pump inhibitory assay. Asterisk stand for HV-CRKP. Source A means strain isolated from sputum; source B means strain isolated from urine; source C means strain isolated from blood; source D means strain isolated from secretion. Hospital a is Wanzhou Three Gorges Central Hospital; hospital b is Fengdu People’s Hospital; hospital c is Qianjiang Central Hospital; hospital d is Jiangjin Central Hospital; hospital e is Dianjiang People’s Hospital; hospital f is Youyang County People’s Hospital; hospital g is Chongqing Ninth People’s Hospital; hospital h is Qijiang District People’s Hospital; and hospital i is People’s Hospital of Banan District.

As shown in Figure 1 and Table 2, 90.2% (46/51) CRKP strains were detected as carbapenemase producers: 70.6% (36/51) isolates possessed *bla*_KPC–2_, 21.6% (11/51) isolated contained *bla*_NDM–1_, 7.8% (4/51) isolates carried *bla*_NDM–5_, and 3.9% (2/51) isolates had *bla*_IMP–4_. Notably, the proportion of strains co-expressing carbapenemases was 11.8% (6/51): four isolates co-carrying *bla*_KPC–2_ and *bla*_NDM–1_, one isolate co-harboring *bla*_KPC–2_ and *bla*_IMP–4_, and one isolate co-carrying *bla*_KPC–2_, *bla*_NDM–1_, and *bla*_IMP–4_. In addition to the production of carbapenemase, 100% (51/51) and 15.7% (8/51) of the CRKP isolates were positive for *ESBLs* and *AmpC* genes, respectively. The *bla*_SHV_ type (72.5%, 37/51) and *bla*_CTX–M–9_ type (66.7%, 34/51) were the most prevalent among CRKP isolates carrying *ESBLs*. Additionally, fluoroquinolone and aminoglycoside genes were detected in 82.4% (42/51) and 88.2% (45/51) of all isolates, with *qnrS* (37/42) and *rmtB* (36/45) being the most common, respectively. In all of these isolates, only two isolates lost both *ompK*35 and *ompK*36 porins and one isolate lost *ompK*36 porin. Moreover, the MICs of ertapenem were observed to have at least a fourfold decrease in the presence of PAβN in 3.9% (2/51) of the CRKP isolates.

**TABLE 2:** Distribution and corresponding carbapenem MIC ranges for CRKP strains with different resistance determinants.

Carbapenem resistance mechanisms	Number of isolates	MIC range (mg/L)		
		ETP	IMP	MEM
**Carbapenemase positive (*n* = 46)**				
*bla*_KPC–2_, no loss OMPs	30	8–512	4–512	4–256
*bla*_KPC–2_, + *bla*_NDM–1_, no loss OMPs	3	256–512	32–128	64–128
*bla*_KPC–2_, + *bla*_NDM–1_, loss OMPs	1	256	128	128
*bla*_KPC–2_, + *bla*_IMP–4_, no loss OMPs	1	256	64	128
*bla*_KPC–2_, + *bla*_NDM–1_ + *bla*_IMP–4_, no loss OMPs	1	32	64	32
*bla*_NDM–1_, no loss OMPs	6	8–64	8–32	4–8
*bla*_NDM–5_, no loss OMPs	2	64–256	32	64
*bla*_NDM–5_, loss OMPs	2	64–128	32–64	32–64
**Carbapenemase negative (*n* = 5)**				
*ESBLs*, no loss OMPs	1	64	16	16
*ESBLs*, *AmpC*, no loss OMPs	2	4–64	0.5–32	0.5–16
*ESBLs*, no loss OMPs, Efflux pump	2	4	0.5–1	0.5

OMPs, outer membrane proteins; ETP, ertapenem; IPM, imipenem; MEM, meropenem.

### Molecular epidemiology of CRKP isolates

**FIGURE 2:**
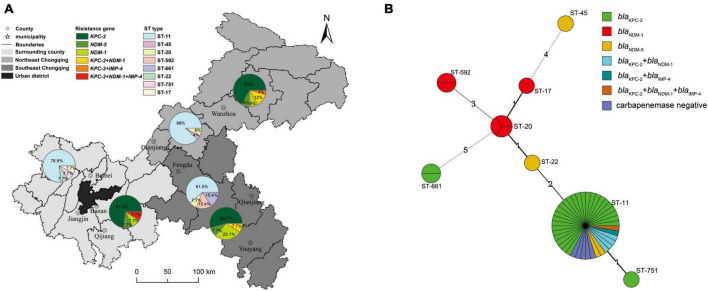
Geographical carbapenemase-producing gene and MLST characteristics of 51 CRKP isolates. **(A)** The nine district and county-level hospitals were divided into northeastern Chongqing, southeastern Chongqing and peripheral regions of the main city according to their administrative areas, and different regions were represented by different background colors. The pie charts represent the prevalence of CRKP strains carrying carbapenemase genes and ST typing in different regions. **(B)** Minimum spanning tree of 51 CRKP isolates based on MLST. In this figure, each circle represents an MLST type, the size of the circle represents the number of strains contained in the type, the number around the circle represents the MLST type, the length of the line between the two circles and the number on the connecting line represents the number of points that the two types differ from each other, and different colors represent strains with different characteristics.

The detailed characteristics of the molecular epidemiology of the CRKP strains were displayed in Figures 1, 2B. A total of 51 CRKP strains were grouped into eight types by MLST method: ST11 was the most prevalent genotype (40/51, 78.4%), followed by ST20 (3/51, 5.9%), ST592 (2/51, 3.9%), and ST661 (2/5, 3.9%), and the other four types only contained one strain. Thirty-six CRKP isolates carrying *bla*_KPC–2_ contained three distinct MLST types, with ST11 being the predominant ST (33/36, 91.7%). Six HV-CRKP isolates belonged to ST11 (*n* = 3), ST592 (*n* = 2), and ST17 (*n* = 1), respectively. Additionally, the molecular typing of all the CRKP isolates was grouped into 29 different PFGE patterns, and 40 ST11 isolates belonged to 20 different PFGE clusters. Meanwhile, the similar PFGE patterns existed in CRKP strains from different hospitals, indicating that there might be clonal transmission of CRKP among hospitals in different regions. Particularly, PFGE cluster 12 contains CRKP strains from four hospitals. The six HV-CRKP strains belonged to six different PFGE patterns. Interestingly, the highly virulent CRKP-31 and CRKP-34 strains isolated from hospital-6 exhibited high similarity of PFGE patterns, suggesting the possibility of nosocomial clonal transmission.

The geographical distribution characteristics of CRKP strains were shown in Figure 2A. The nine district-level hospitals were divided into three major regions: surrounding county, northeast Chongqing, and southeast Chongqing according to the geographical distance and orientation from the central urban area. Obviously, the ST11 genotype was the predominant ST type in all three regions. The percentage of ST11 in descending order was in northeast Chongqing (88.0%), the surrounding county (76.9%), and southeast Chongqing (61.5%), while other ST types were only scattered distribution.

For the distribution of the carbapenemase-producing genes, the proportion of carbapenemase-producing CRKP strains isolated in northeastern Chongqing was higher than that in southeastern Chongqing and the surrounding county, with percentages of 96, 84.7, and 84.6%, respectively. Carbapenemase-producing CRKP strains mainly carried the *bla*_KPC_ gene, but the *bla*_NDM_ carriage rate of CRKP strains in southeast Chongqing was significantly higher than that in northeast Chongqing and surrounding areas, with the proportions of 38.5, 28.0, and 23.1%, respectively. Alarmingly, the rate of CRKP strains carrying more than 2 carbapenemases was higher in northeast Chongqing than in the other two regions. Four of the six HV-CRKP strains were isolated from southeast Chongqing and two from the surrounding county. These results suggested that those district hospitals far from the central urban area showed a higher proportion of carbapenemases-positive and highly virulent CRKP strains.

### Detection of capsular serotyping, virulence-associated determinants, and serum killing assay

Among the 51 CRKP isolates, 11.8% (6/51) CRKP isolates were positive for the string test and defined as HV-CRKP, which were separated from urine (*n* = 3), sputum (*n* = 2), and blood (*n* = 1). Genotyping of the six HV-CRKP strains revealed that two isolates belonged to the K57 serotype, but the other four strains were K-nontypeable, which was not classified in any of the K1, K2, K5, K20, K54, or K57 serotype.

**FIGURE 3:**
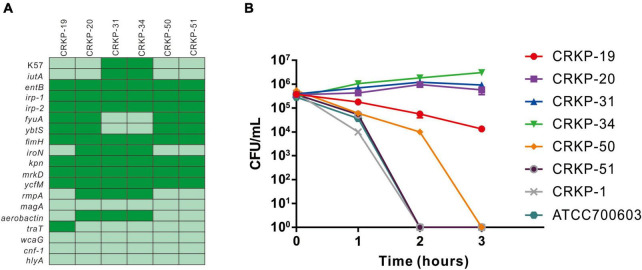
Virulence gene and serum killing assay analysis of CRKP strains. **(A)** The presence of virulence genes in a specific genome is represented by the dark green box and the absence of virulence genes is represented by a light green box. **(B)** Serum killing assay of six hypermucoviscous CRKP strains. Survival of each strain was assessed by enumerating viable counts at 0, 1, 2, and 3 h of incubation in the pooled human sera at 37°C. Data are mean ± SEM (*n* = 3 for each strain).

The prevalence of virulence-associated genes among HV-CRKP isolates was listed in Figure 3A. All HV-CRKPs harbored the virulence-associated genes such as *entB*, *irp-1/2*, *fimH*, *kpn*, *mrkD*, and *ycfM*. The most important virulence genes for HV-CRKP including *rmpA*, *aerobactin*, and *iroN* were detected in three isolates. The *iutA* gene was detected in CRKP-31 and CRKP-34 strains, and the other four isolates carried *fyuA* and *ybtS* genes. The remaining virulence-associated genes *magA*, *wcaG*, *cnf-1*, and *hlyA* genes were not detected in any of the HV-CRKP isolates. Serum killing resistance was found in CRKP-20, CRKP-31, and CRKP-34 isolates. The CRKP-19 strain showed intermediately sensitive, the other two HV-CRKP isolates exhibited complete sensitivity (Figure 3B).

## Discussion

The emergence of CRKP has increased rapidly in the past decade and has become a global public health problem. Not only that but there are also distinct differences in the molecular epidemiology and drug resistance mechanisms of CRKP in different continents, countries, and regions (Hu et al., 2020), especially developing countries and regions showing higher levels of carbapenem antibiotic resistance (Logan and Weinstein, 2017). Numerous studies have focused on CRKP prevalence in urban cities or large teaching hospitals (Zhan et al., 2017; Tang et al., 2020), however CRKP epidemiology in regional or non-teaching hospitals has been overlooked. In this study, we investigated the molecular prevalence characteristics and resistance mechanisms of CRKP for the first time in district-level hospitals in Chongqing, Southwest China.

Our results showed that CRKP strains in district hospitals presented high levels of MIC_50_ and MIC_90_ in carbapenem antibiotics especially ertapenem and meropenem compared with Chongqing urban hospitals (Yan et al., 2017), which was probably related to the irrational application of antibiotics in district hospitals (Taxifulati et al., 2021; Ayobami et al., 2022). On the other hand, another district-teaching hospital in Chongqing also reported that the high level of carbapenem MIC_50_ and MIC_90_ for CRKP strains (Zeng et al., 2021), emphasized the horizontal and clonal transmission of the carbapenemase gene. The CRKP strains isolated from 17 district-level hospitals in Nanjing, China displayed high MIC_50_ and MIC_90_ for almost all clinically available antibiotics, including carbapenems (Zhou et al., 2020). Similarly, the mechanism of CRKP resistance in this study was also dominated by the carbapenemase-producing mechanism consistent with the above-mentioned district hospitals’ study. Our results showed that CRKP strains isolated from district hospitals carried a high rate of the *bla*_KPC_ (70.6%) gene, especially a high prevalence of the *bla*_NDM_ gene (29.4%). Carbapenemase-producing strains exhibited higher cutoff values of carbapenem MIC than non-carbapenemase-producing strains including *Enterobacteriaceae*, *Pseudomonas*, and *Acinetobacter* isolates (Tamma et al., 2016, 2017b). The proportion of CRKP strains co-expressing carbapenemase genes reached 11.8% in our study, with 100% *ESBLs* gene carriage, 82.4% fluoroquinolone resistance gene carriage, and 88.2% aminoglycoside resistance gene carriage. More interestingly, a strain carrying *bla*_KPC–2_, *bla*_NDM–1_, and *bla*_IMP–4_ genes was isolated for the first time, but its MIC value to carbapenem antibiotics did not increase significantly, probably due to the low expression of these carbapenemases. Double- or multi-carbapenemase producers have been reported worldwide in different strains, however, the influence on MICs value needs further investigation (Meletis et al., 2015; Niu et al., 2020). Previous research has indeed established that non-carbapenemase-producing CRE may be attributed to alterations or deletions in outer membrane proteins, coupled with the overexpression of cephalosporinase, the production of ESBLs, and the upregulation of efflux pumps (Liu et al., 2021; Zhang et al., 2022). However, the correlation between outer membrane proteins (OMPs) and carbapenemase genes in bacteria has been rarely investigated. Theoretically, the combined impact of OMPs deletion and carbapenemase production could lead to a higher level of resistance to carbapenem antibiotics than either mechanism alone, and potentially contributing to multidrug resistance. OMPs play a pivotal role in controlling bacterial outer membrane permeability, affecting susceptibility to antibiotics, including carbapenems. On the other hand, carbapenemase genes can hydrolyze carbapenem antibiotics, rendering them ineffective (Ma et al., 2023). OMP alterations can synergize with the presence of carbapenemase genes to enhance carbapenem resistance. However, our study did not yield evidence supporting such a correlation. This discrepancy might be attributed to our focus solely on the deletion of OMPs, without considering OMPs mutations or expression levels, or it could be influenced by our relatively small sample size. This intriguing observation aligns with findings in our previously published research, highlighting the need for further in-depth investigation (Jia et al., 2018). The complex interactions between carbapenemase and OMPs require further exploration.

*Klebsiella pneumoniae* stands as one of well-established culprits in hospital-acquired infections and is particularly notorious for inciting outbreaks within healthcare settings. This predisposition has been instrumental in the successful spread of CRKP (Effah et al., 2020). Prolonged hospital stays and extended antibiotic treatments can create favorable conditions for *K. pneumoniae* colonization in the gastrointestinal tract and oropharynx, heightening patients’ vulnerability to infections originating from their own microbiota. Notably, CRE gut colonized patients could reemerge and promote systemic infection even after antibiotic cessation, and further contributing to nosocomial transmission (Korach-Rechtman et al., 2020). Consistent with previous studies, CRKP was prone to infect infants and the elderly and linked with poorer clinical prognosis (Tamma et al., 2017a; Hu et al., 2020). Infants and young children have developing immune systems that are not yet fully mature, making them vulnerable to a variety of pathogens (Bor and Ilhan, 2021). The immune system of the elderly usually declines with age, and their immune function declines, making them vulnerable to CRKP infection (Hu et al., 2020). In our study, these CRKP strains still keep high sensitivity to tigecycline and colistin, which has been reported that the application of tigecycline and/or colistin agents would obtain an effective clearance of CRKP bacteremia both *in vivo* and *in vitro* (Tamma et al., 2017a; Fergadaki et al., 2021). Tigecycline is a protein synthesis inhibitor that inhibits protein synthesis by binding to the 30S subunit of bacterial ribosomes (Yaghoubi et al., 2022). Colistin is a lipopolysaccharide antibiotic that causes cell death by damaging bacterial cell membranes (El-Sayed Ahmed et al., 2020). Its unique bactericidal mechanism and destruction of bacterial membranes make colistin effective even against bacteria with high resistance to other antibiotics. In the present study, CRKP mainly acquires resistance to carbapenem antibiotics by producing carbapenemase, an enzyme that degrades carbapenem antibiotics. However, colistin and tigecycline are not susceptible to carbapenemase degradation and therefore may apply to treatment of clinical CRKP infection.

Our study showed that CRKP strains in district hospitals were similarly dominated by the ST11 type (78.4%), which was consistent with Chinese large urban teaching hospitals and other regional hospitals (Zhou et al., 2020; Zeng et al., 2021). CRKP was predominantly prevalent in Europe and the United States with ST258, while China and South America were dominated by ST11 (Wang et al., 2022). ST11 is a tonB single-gene variant of ST258, both of which originated from the clonal complex CC258 (Guo et al., 2022). Meanwhile, 82.5% of the isolated ST11-type CRKP strains carried the *bla*_KPC–2_ gene. ST11-*bla*_KPC–2_-CRKP has been reported as one of the most dominant genotypes in China (Hu et al., 2020; Guo et al., 2022). In our study, 11.8% of CRKP strains were identified as HV-CRKP strains, similar to the previously reported prevalence of 12.1% (Yao et al., 2015). Along with the global dissemination of mobile genetic elements conferring antibiotic resistance or virulence, carbapenem-resistant hypervirulent *K. pneumoniae* or hypervirulent carbapenem-resistant *K. pneumoniae* increased rapidly, especially hypervirulent and carbapenem-resistant ST11 *K. pneumoniae* strains (Yao et al., 2015; Zhan et al., 2017). Capsular serotyping and serum killing assay showed that the ST11 HV-CRKP strains were K-nontypeable and showed high serum resistance, which also carried both *rmpA* and *aerobactin* virulent genes (Yao et al., 2015). HV-CRKP ST11 strain has been confirmed to lead to increased mortality in hospitalized patients, prolonged hospitalization, and nosocomial transmission, which substantially threatened human health and needed great attention (Gu et al., 2018; Huang J. et al., 2022; Huang N. et al., 2022). Interestingly, we isolated two HV-CRKP strains of ST592 for the first time. PFGE homology analysis showed that these two virulent strains were highly similar, and the possibility of nosocomial clonal transmission existed.

The distribution map of CRKP strains showed that the strains isolated from different regions had some differences in the ratio of carbapenemase production, carbapenemase classification, and ST type. The percentage of carbapenemase-producing strains and the percentage of ST11 type were the highest in Northeast Chongqing, the *bla*_NDM_ gene carriage rate of strains isolated in Southeast Chongqing was higher than the other two regions. The geographical distribution of CRKP strains in different countries and regions is not exactly the same (Zhang et al., 2016; Wang et al., 2022; Wyres and Holt, 2022). Hu et al. (2020) reported that the prevalence of CRKP in Chinese coastal cities was higher than that in mountainous areas. However, the generation of this difference still needed further research, which was possibly related to local medical conditions, antibiotic use habits, and economic development (Taxifulati et al., 2021; Ayobami et al., 2022). The PFGE results demonstrated that some similar clones came from different district hospitals, suggesting the existence of cross-regional clone transmission, which may become one of the key points to preventing CRKP dissemination.

This study has some strengths and limitations. First, CRKP strains were only collected for one year, and the amount of these strains was also small, which restricted the conclusion applied in other Chinese cities. However, this study collected representative CRKP strains from nine district hospitals and nearly covered all districts in Chongqing. Secondly, we did not deeply explore the plasmid typing, because our other study would further focus on the mechanism of resistance and hypervirulent genes transmission by using whole genome sequencing, especially for the co-expressing *bla*_KPC–2_, *bla*_NDM–1_, and *bla*_IMP–4_ isolate. Nevertheless, this was the first comprehensive study to investigate the dissemination and characteristics of CRKP in nine district hospitals in southwestern China and would provide support for the prevention and control of nosocomial infection by CRKP in secondary hospitals in the future.

## Conclusion

Carbapenem-resistant *K. pneumoniae* strains in district hospitals of Chongqing showed epidemic characteristics of high MIC values, a high proportion of carbapenemase production, co-expression of dual or multiple carbapenemases, and virulent strains. Clonal transmissions of CRKP strains and HV-CRKP strains have occurred in intra-hospital and interregional transmission among different hospitals, which should cause great concern and take effective corresponding measures.

## Data availability statement

The raw data supporting the conclusions of this article will be made available by the authors, without undue reservation.

## Ethics statement

The studies involving humans were approved by the Biomedical Ethics Committee of the First Affiliated Hospital of Chongqing Medical University. The studies were conducted in accordance with the local legislation and institutional requirements. Written informed consent for participation in this study was provided by the participants’ legal guardians/next of kin.

## Author contributions

HL: Data curation, Writing – original draft, Writing – review and editing. YW: Data curation, Funding acquisition, Writing – original draft, Writing – review and editing, Methodology. YO: Formal analysis, Methodology, Writing – review and editing. XX: Data curation, Methodology, Writing – review and editing. SS: Data curation, Methodology, Writing – review and editing. XT: Data curation, Methodology, Writing – review and editing. YX: Funding acquisition, Supervision, Writing – review and editing.

## Footnotes

1
https://bigsdb.pasteur.fr/klebsiella/

## References

[B1] Ayobami O. Brinkwirth S. Eckmanns T. Markwart R. (2022). Antibiotic resistance in hospital-acquired ESKAPE-E infections in low- and lower-middle-income countries: A systematic review and meta-analysis. *Emerg. Microbes Infect.* 11 443–451. 10.1080/22221751.2022.2030196 35034585 PMC8820817

[B2] Bor M. Ilhan O. (2021). Carbapenem-resistant *Klebsiella pneumoniae* outbreak in a neonatal intensive care unit: Risk factors for mortality. *J. Trop. Pediatr.* 67:fmaa057. 10.1093/tropej/fmaa057 32778897

[B3] CLSI (2021). *Performance standards for antimicrobial susceptibility testing, M100, 31st ed.* Wayne, PA: Clinical and Laboratory Standards Institute. 10.1128/JCM.00213-21 PMC8601225 34550809

[B4] Effah C. Y. Sun T. Liu S. Wu Y. (2020). *Klebsiella pneumoniae*: An increasing threat to public health. *Ann. Clin. Microbiol. Antimicrob.* 19 1. 10.1186/s12941-019-0343-8 31918737 PMC7050612

[B5] El-Sayed Ahmed M. A. E.-G. Zhong L.-L. Shen C. Yang Y. Doi Y. Tian G.-B. (2020). Colistin and its role in the Era of antibiotic resistance: an extended review (2000-2019). *Emerg. Microbes Infect.* 9 868–885. 10.1080/22221751.2020.1754133 32284036 PMC7241451

[B6] Fergadaki S. Renieris G. Machairas N. Sabracos L. Droggiti D.-I. Misiakos E. (2021). Efficacy of tigecycline alone or in combination for experimental infections by KPC carbapenemase-producing *Klebsiella pneumoniae*. *Int. J. Antimicrob. Agents* 58:106384. 10.1016/j.ijantimicag.2021.106384 34161789

[B7] Gu D. Dong N. Zheng Z. Lin D. Huang M. Wang L. (2018). A fatal outbreak of ST11 carbapenem-resistant hypervirulent *Klebsiella pneumoniae* in a Chinese hospital: A molecular epidemiological study. *Lancet Infect. Dis.* 18 37–46. 10.1016/S1473-3099(17)30489-9 28864030

[B8] Guo L. Wang L. Zhao Q. Ye L. Ye K. Ma Y. (2022). Genomic analysis of KPC-2-producing *Klebsiella pneumoniae* ST11 isolates at the respiratory department of a tertiary care hospital in Beijing, China. *Front. Microbiol.* 13:929826. 10.3389/fmicb.2022.929826 35783384 PMC9244631

[B9] Hu Y. Liu C. Shen Z. Zhou H. Cao J. Chen S. (2020). Prevalence, risk factors and molecular epidemiology of carbapenem-resistant *Klebsiella pneumoniae* in patients from Zhejiang, China, 2008-2018. *Emerg. Microbes Infect.* 9 1771–1779. 10.1080/22221751.2020.1799721 32689907 PMC7475806

[B10] Huang J. Yi M. Yuan Y. Xia P. Yang B. Liao J. (2022). Emergence of a fatal ST11-KL64 tigecycline-resistant hypervirulent *Klebsiella pneumoniae* clone cocarrying blaNDM and blaKPC in Plasmids. *Microbiol. Spectr.* 10 e0253922. 10.1128/spectrum.02539-22 36205391 PMC9769963

[B11] Huang N. Jia H. Zhou B. Zhou C. Cao J. Liao W. (2022). Hypervirulent carbapenem-resistant *Klebsiella pneumoniae* causing highly fatal meningitis in southeastern China. *Front Public Health* 10:991306. 10.3389/fpubh.2022.991306 36324461 PMC9621088

[B12] Jia X. Dai W. Ma W. Yan J. He J. Li S. (2018). Carbapenem-Resistant *E. cloacae* in Southwest China: Molecular analysis of resistance and risk factors for infections caused by NDM-1-producers. *Front. Microbiol.* 9:658. 10.3389/fmicb.2018.00658 29670607 PMC5893741

[B13] Korach-Rechtman H. Hreish M. Fried C. Gerassy-Vainberg S. Azzam Z. S. Kashi Y. (2020). Intestinal dysbiosis in carriers of carbapenem-resistant *Enterobacteriaceae*. *mSphere* 5 e173–e120. 10.1128/mSphere.00173-20 32350099 PMC7193040

[B14] Lan P. Jiang Y. Zhou J. Yu Y. (2021). A global perspective on the convergence of hypervirulence and carbapenem resistance in *Klebsiella pneumoniae*. *J. Glob. Antimicrob. Resist.* 25 26–34. 10.1016/j.jgar.2021.02.020 33667703

[B15] Lau M. Y. Teng F. E. Chua K. H. Ponnampalavanar S. Chong C. W. Abdul Jabar K. (2021). Molecular characterization of carbapenem resistant *Klebsiella pneumoniae* in Malaysia Hospital. *Pathogens* 10:279. 10.3390/pathogens10030279 33801250 PMC8001961

[B16] Lee H. C. Chuang Y. C. Yu W. L. Lee N. Y. Chang C. M. Ko N. Y. (2006). Clinical implications of hypermucoviscosity phenotype in *Klebsiella pneumoniae* isolates: Association with invasive syndrome in patients with community-acquired bacteraemia. *J. Intern. Med.* 259 606–614. 10.1111/j.1365-2796.2006.01641.x 16704562

[B17] Liu H. Jia X. Zou H. Sun S. Li S. Wang Y. (2019). Detection and characterization of tigecycline heteroresistance in *E. cloacae*: Clinical and microbiological findings. *Emerg. Microbes Infect.* 8 564–574. 10.1080/22221751.2019.1601031 30945610 PMC6455127

[B18] Liu S. Huang N. Zhou C. Lin Y. Zhang Y. Wang L. (2021). Molecular mechanisms and epidemiology of carbapenem-resistant *Enterobacter cloacae* Complex isolated from Chinese Patients During 2004-2018. *Infect. Drug Resist.* 14 3647–3658. 10.2147/IDR.S327595 34522107 PMC8434891

[B19] Logan L. K. Weinstein R. A. (2017). The epidemiology of carbapenem-resistant *Enterobacteriaceae*: The impact and evolution of a global menace. *J. Infect. Dis.* 215 S28–S36. 10.1093/infdis/jiw282 28375512 PMC5853342

[B20] Luterbach C. L. Chen L. Komarow L. Ostrowsky B. Kaye K. S. Hanson B. (2023). Transmission of Carbapenem-Resistant *Klebsiella pneumoniae* in US Hospitals. *Clin. Infect. Dis.* 76 229–237. 10.1093/cid/ciac791 36173830 PMC10202433

[B21] Ma J. Song X. Li M. Yu Z. Cheng W. Yu Z. (2023). Global spread of carbapenem-resistant *Enterobacteriaceae*: Epidemiological features, resistance mechanisms, detection and therapy. *Microbiol. Res.* 266 127249. 10.1016/j.micres.2022.127249 36356348

[B22] Meletis G. Chatzidimitriou D. Malisiovas N. (2015). Double- and multi-carbapenemase-producers: the excessively armored bacilli of the current decade. *Eur. J. Clin. Microbiol. Infect. Dis.* 34 1487–1493. 10.1007/s10096-015-2379-9 25894987

[B23] Niu S. Wei J. Zou C. Chavda K. D. Lv J. Zhang H. (2020). In vitro selection of aztreonam/avibactam resistance in dual-carbapenemase-producing *Klebsiella pneumoniae*. *J. Antimicrob. Chemother.* 75 559–565. 10.1093/jac/dkz468 31722380 PMC7021086

[B24] Nordmann P. Poirel L. (2019). Epidemiology and diagnostics of carbapenem resistance in gram-negative Bacteria. *Clin. Infect. Dis.* 69 S521–S528. 31724045 10.1093/cid/ciz824 PMC6853758

[B25] Soto E. Marchi S. Beierschmitt A. Kearney M. Francis S. VanNess K. (2016). Interaction of non-human primate complement and antibodies with hypermucoviscous *Klebsiella pneumoniae*. *Vet. Res.* 47 40. 10.1186/s13567-016-0325-1 26951091 PMC4782414

[B26] Ssekatawa K. Byarugaba D. K. Nakavuma J. L. Kato C. D. Ejobi F. Tweyongyere R. (2021). Prevalence of pathogenic *Klebsiella pneumoniae* based on PCR capsular typing harbouring carbapenemases encoding genes in Uganda tertiary hospitals. *Antimicrob. Resist. Infect. Control* 10 57. 10.1186/s13756-021-00923-w 33736698 PMC7977577

[B27] Tamma P. D. Goodman K. E. Harris A. D. Tekle T. Roberts A. Taiwo A. (2017a). Comparing the outcomes of patients with carbapenemase-producing and non-carbapenemase-producing carbapenem-resistant *Enterobacteriaceae* Bacteremia. *Clin. Infect. Dis.* 64 257–264. 10.1093/cid/ciw741 28013264 PMC5241781

[B28] Tamma P. D. Huang Y. Opene B. N. A. Simner P. J. (2016). Determining the optimal carbapenem MIC that distinguishes carbapenemase-producing and non-carbapenemase-producing carbapenem-resistant *Enterobacteriaceae*. *Antimicrob. Agents Chemother.* 60 6425–6429. 10.1128/AAC.00838-16 27503655 PMC5038245

[B29] Tamma P. D. Wang R. Lewis S. Opene B. N. A. Simner P. J. (2017b). Is there a carbapenem MIC cutoff value that distinguishes carbapenemase-producing and non-carbapenemase-producing carbapenem non-susceptible *Pseudomonas* and acinetobacter isolates? *Infect. Control Hosp. Epidemiol.* 38 1378–1379. 10.1017/ice.2017.210 28965496 PMC5748885

[B30] Tang Y. Liu H. Zhao J. Yi M. Yuan Y. Xia Y. (2020). Clinical and microbiological prognostic factors of in-hospital mortality caused by hypervirulent *Klebsiella pneumoniae* infections: A retrospective study in a tertiary hospital in Southwestern China. *Infect. Drug Resist.* 13 3739–3749. 10.2147/IDR.S276642 33116694 PMC7586058

[B31] Taxifulati Y. Wushouer H. Fu M. Zhou Y. Du K. Zhang X. (2021). Antibiotic use and irrational antibiotic prescriptions in 66 primary healthcare institutions in Beijing City, China, 2015-2018. *BMC Health Serv. Res.* 21:832. 10.1186/s12913-021-06856-9 34404405 PMC8371863

[B32] Tenover F. C. Arbeit R. D. Goering R. V. Mickelsen P. A. Murray B. E. Persing D. H. (1995). Interpreting chromosomal DNA restriction patterns produced by pulsed-field gel electrophoresis: criteria for bacterial strain typing. *J. Clin. Microbiol.* 33 2233–2239. 10.1128/jcm.33.9.2233-2239.1995 7494007 PMC228385

[B33] Wang M. Earley M. Chen L. Hanson B. M. Yu Y. Liu Z. (2022). Clinical outcomes and bacterial characteristics of carbapenem-resistant *Klebsiella pneumoniae* complex among patients from different global regions (CRACKLE-2): a prospective, multicentre, cohort study. *Lancet Infect. Dis.* 22 401–412. 10.1016/S1473-3099(21)00399-6 34767753 PMC8882129

[B34] Wyres K. Holt K. (2022). Regional differences in carbapenem-resistant *Klebsiella pneumoniae*. *Lancet Infect. Dis.* 22 309–310. 10.1016/S1473-3099(21)00425-4 34767752

[B35] Yaghoubi S. Zekiy A. O. Krutova M. Gholami M. Kouhsari E. Sholeh M. (2022). Tigecycline antibacterial activity, clinical effectiveness, and mechanisms and epidemiology of resistance: narrative review. *Eur. J. Clin. Microbiol. Infect. Dis.* 41 1003–1022. 10.1007/s10096-020-04121-1 33403565 PMC7785128

[B36] Yan J. Pu S. Jia X. Xu X. Yang S. Shi J. (2017). Multidrug resistance mechanisms of carbapenem resistant *Klebsiella pneumoniae* strains isolated in Chongqing, China. *Ann. Lab. Med.* 37 398–407. 10.3343/alm.2017.37.5.398 28643488 PMC5500738

[B37] Yang X. Sun Q. Li J. Jiang Y. Li Y. Lin J. (2022). Molecular epidemiology of carbapenem-resistant hypervirulent *Klebsiella pneumoniae* in China. *Emerg. Microbes Infect.* 11 841–849. 10.1080/22221751.2022.2049458 35236251 PMC8942559

[B38] Yao B. Xiao X. Wang F. Zhou L. Zhang X. Zhang J. (2015). Clinical and molecular characteristics of multi-clone carbapenem-resistant hypervirulent (hypermucoviscous) *Klebsiella pneumoniae* isolates in a tertiary hospital in Beijing, China. *Int. J. Infect. Dis.* 37 107–112. 10.1016/j.ijid.2015.06.023 26141415

[B39] Zeng L. Yang C. Zhang J. Hu K. Zou J. Li J. (2021). An outbreak of carbapenem-resistant *Klebsiella pneumoniae* in an intensive care unit of a major teaching hospital in Chongqing, China. *Front. Cell Infect. Microbiol.* 11:656070. 10.3389/fcimb.2021.656070 34150672 PMC8208809

[B40] Zhan L. Wang S. Guo Y. Jin Y. Duan J. Hao Z. (2017). Outbreak by hypermucoviscous *Klebsiella pneumoniae* ST11 isolates with carbapenem resistance in a tertiary hospital in China. *Front. Cell Infect. Microbiol.* 7:182. 10.3389/fcimb.2017.00182 28560183 PMC5432538

[B41] Zhang H. Wang J. Zhou W. Yang M. Wang R. Yan X. (2021). Risk factors and prognosis of carbapenem-resistant *Klebsiella pneumoniae* infections in respiratory intensive care unit: A Retrospective Study. *Infect. Drug Resist.* 14 3297–3305. 10.2147/IDR.S317233 34447257 PMC8382964

[B42] Zhang K. Liu L. Yang M. Chen C. Li X. Tian J. (2022). Reduced porin expression with EnvZ-OmpR, PhoPQ, BaeSR two-component system down-regulation in carbapenem resistance of *Klebsiella Pneumoniae* based on proteomic analysis. *Microb. Pathog.* 170 105686. 10.1016/j.micpath.2022.105686 35917986

[B43] Zhang Y. Wang Q. Yin Y. Chen H. Jin L. Gu B. (2018). Epidemiology of Carbapenem-Resistant *Enterobacteriaceae* Infections: Report from the China CRE Network. *Antimicrob. Agents Chemother.* 62 e1882–e1817. 10.1128/AAC.01882-17 29203488 PMC5786810

[B44] Zhang Y. Zhao C. Wang Q. Wang X. Chen H. Li H. (2016). High prevalence of hypervirulent *Klebsiella pneumoniae* Infection in China: Geographic distribution, clinical characteristics, and antimicrobial resistance. *Antimicrob. Agents Chemother.* 60 6115–6120. 10.1128/AAC.01127-16 27480857 PMC5038323

[B45] Zhen X. Stålsby Lundborg C. Sun X. Gu S. Dong H. (2020). Clinical and economic burden of carbapenem-resistant infection or colonization caused by *Klebsiella pneumoniae, Pseudomonas aeruginosa, Acinetobacter baumannii*: A Multicenter Study in China. *Antibiotics* 9 514. 10.3390/antibiotics9080514 PMC7459498 32823707

[B46] Zhou H. Zhang K. Chen W. Chen J. Zheng J. Liu C. (2020). Epidemiological characteristics of carbapenem-resistant *Enterobacteriaceae* collected from 17 hospitals in Nanjing district of China. *Antimicrob. Resist Infect. Control* 9 15. 10.1186/s13756-019-0674-4 31956404 PMC6958626

[B47] Zong Z. Wu A. Hu B. (2020). Infection control in the era of antimicrobial resistance in China: Progress, Challenges, and opportunities. *Clin. Infect. Dis.* 71 S372–S378. 10.1093/cid/ciaa1514 33367579

[B48] Zou H. Jia X. Liu H. Li S. Wu X. Huang S. (2020). Emergence of NDM-5-Producing *Escherichia coli* in a Teaching Hospital in Chongqing, China: IncF-Type Plasmids May Contribute to the Prevalence of bla NDM- 5. *Front. Microbiol.* 11:334. 10.3389/fmicb.2020.00334 32210935 PMC7069339

