# Supplementary material for: Dissemination and characteristics of carbapenem-resistant Klebsiella pneumoniae in nine district hospitals in southwestern China
Source: Front Microbiol. 2023 Oct 24;14:1269408. doi: 10.3389/fmicb.2023.1269408 (PMC10628634; doi:10.3389/fmicb.2023.1269408)
Supplement: Supplementary file 1 [file Table_1.doc]

**Supplementary Table 1** Primer sequences of resistant genes, virulence genes and housekeeping gene for MLST alleles

| **Target gene** | **Primer** | **Primer sequence（5’-3’）** | **Product size（bp）** | **Temperature（℃）** |
| --- | --- | --- | --- | --- |
| Resistant genes | | | | |
| *bla_KPC_* | F | AACCATTCGCTAAACTCG | 781 | 50 |
|  | R | TTGACGCCCAATCCC |  |  |
| *bla_NDM_* | F | GGTTTGGCGATCTGGTTTTC | 621 | 55 |
|  | R | CGGAATGGCTCATCACGATC |  |  |
| *bla_IMP_* | F | CTACCGCAGCAGAGTCTTTG | 587 | 52 |
|  | R | AACCAGTTTTGCCTTACCAT |  |  |
| *bla_VIM_* | F | TCGCATATCGCAACGCAG | 500 | 58 |
|  | R | GTGGGCCATTCAGCCAGA |  |  |
| *bla_OXA-48_* | F | CCGACCCACCAGCCAATCT | 494 | 60 |
|  | R | AAACGGGCGAACCAAGCA |  |  |
| *bla_CTX-M-1_* | F | AAGACTGGGTGTGGCATTGA | 670 | 55 |
|  | R | AGGCTGGGTGAAGTAAGTGA |  |  |
| *bla_CTX-M-9_* | F | GCTTTATGCGCAGACGAGTG | 686 | 55 |
|  | R | GCCAGATCACCGCAATATCA |  |  |
| *bla_SHV_* | F | CTTTACTCGCCTTTATCGGC | 1031 | 60 |
|  | R | TTACCGACCGGCATCTTTCC |  |  |
| *bla_TEM_* | F | GTGCGCGGAACCCCTATT | 919 | 60 |
|  | R | TTACCAATGCTTAATCAGTGAGGC |  |  |
| *qnrA* | F | ATTTCTCACGCCAGGATTTG | 413 | 53 |
|  | R | GAGATTGGCATTGCTCCAGT |  |  |
| *qnrB* | F | GATCGTGAAAGCCAGAAAGG | 469 | 53 |
|  | R | ACGATGCCTGGTAGTTGTCC |  |  |
| *qnrC* | F | GGGTTGTACATTTATTGAATC | 447 | 47 |
|  | R | TCCACTTTACGAGGTTCT |  |  |
| *qnrD* | F | CGAGATCAATTTACGGGGAATA | 582 | 54 |
|  | R | AACAAGCTGAAGCGCCTG |  |  |
| *qnrS* | F | GCAAGTTCATTGAACAGGGT | 428 | 58 |
|  | R | TCTAAACCGTCGAGTTCGGCG |  |  |
| *aac(6’)-Ib-cr* | F | ATATGCGGATCCAATGAGCAACGCAAAAACAAAGTTAG | 544 | 55 |
|  | R | ATAGCGAATTCTTAGGCATCACTGCGTGTTCGCTC |  |  |
| *aac(6’)-Ib* | F | TATGAGTGGCTAAATCGAT | 395 | 55 |
|  | R | CCCGCTTTCTCGTAGCA |  |  |
| *armA* | F | CCGAAATGACAGTTCCTATC | 846 | 55 |
|  | R | GAAAATGAGTGCCTTGGAGG |  |  |
| *rmtB* | F | ATGAACATCAACGATGCCCT | 769 | 55 |
|  | R | CCTTCTGATTGGCTTATCCA |  |  |
| *ompK35* | F | ATGATGAAGCGCAATATTCTGGCAGTGG | 682 | 66 |
|  | R | TCGGCTTTGTCGCCATTGCCGTCA |  |  |
| *ompK36* | F | ATGAAAGTTAAAGTACTGTCCCTC | 1062 | 62 |
|  | R | GTCGTCGGTAGAGATACCGGC |  |  |
| *CIT* | F | TGGCCAGAACTGACAGGCAAA | 462 | 55 |
|  | R | TTTCTCCTGAACGTGGCTGGC |  |  |
| *DHA* | F | AACTTTCACAGGTGTGCTGGGT | 405 | 55 |
|  | R | CCGTACGCATACTGGCTTTGC |  |  |
| Virulence genes | | | | |
| *K1* | F | GTAGGTATTGCAAGCCATGC | 1046 | 55 |
|  | R | GCCCAGGTTAATGAATCCGT |  |  |
| *K2* | F | GACCCGATATTCATACTTGACAGAG | 641 | 57 |
|  | R | CCTGAAGTAAAATCGTAAATAGATGGC |  |  |
| *K5* | F | TGGTAGTGATGCTCGCGA | 280 | 55 |
|  | R | CCTGAACCCACCCCAATC |  |  |
| *K20* | F | CGGTGCTACAGTGCATCATT | 741 | 55 |
|  | R | GTTATACGATGCTCAGTCGC |  |  |
| *K54* | F | CATTAGCTCAGTGGTTGGCT | 885 | 55 |
|  | R | GCTTGACAAACACCATAGCAG |  |  |
| *K57* | F | CTCAGGGCTAGAAGTGTCAT | 1037 | 55 |
|  | R | CACTAACCCAGAAAGTCGAG |  |  |
| *wcaG* | F | GGTTGGGTCAGCAATCGTA | 169 | 53 |
|  | R | ACTATTCCGCCAACTTTTGC |  |  |
| *rmpA* | F | ACTGGGCTACCTCTGCTTCA | 535 | 50 |
|  | R | CTTGCATGAGCCATCTTTCA |  |  |
| *magA* | F | GGTGCTCTTTACATCATTGC | 1282 | 53 |
|  | R | GCAATGGCCATTTGCGTTAG |  |  |
| *aerobactin* | F | GCATAGGCGGATACGAACAT | 556 | 55 |
|  | R | CACAGGGCAATTGCTTACCT |  |  |
| *fimH-1* | F | ATGAACGCCTGGTCCTTTGC | 688 | 55 |
|  | R | GCTGAACGCCTATCCCCTGC |  |  |
| *mrkD* | F | CCACCAACTATTCCCTCGAA | 240 | 52 |
|  | R | ATGGAACCCACATCGACATT |  |  |
| *kpn* | F | GTATGACTCGGGGAAGATTA | 626 | 55 |
|  | R | CAGAAGCAGCCACCACACG |  |  |
| *ycfM* | F | ATCAGCAGTCGGGTCAGC | 160 | 55 |
|  | R | CTTCTCCAGCATTCAGCG |  |  |
| *entB* | F | ATTTCCTCAACTTCTGGGGC | 371 | 57 |
|  | R | AGCATCGGTGGCGGTGGTCA |  |  |
| *iutA* | F | GGCTGGACATCATGGGAACTGG | 300 | 63 |
|  | R | CGTCGGGAACGGGTAGAATCG |  |  |
| *irp-1* | F | TGAATCGCGGGTGTCTTATGC | 238 | 57 |
|  | R | TCCCTCAATAAAGCCCACGCT |  |  |
| *irp-2* | F | AAGGATTCGCTGTTACCGGAC | 287 | 57 |
|  | R | TCGTCGGGCAGCGTTTCTTCT |  |  |
| *ybtS* | F | AGTGGTGCGTTCTGCGTC | 477 | 50 |
|  | R | ATTTCTACATCTGGCGTTA |  |  |
| *fyuA* | F | GCGACGGGAAGCGATGATTTA | 547 | 56 |
|  | R | TAAATGCCAGGTCAGGTCACT |  |  |
| *iroN* | F | AAGTCAAAGCAGGGGTTGCCCG | 665 | 63 |
|  | R | GACGCCGACATTAAGACGCAG |  |  |
| *trat* | F | GGTGTGGTGCGATGAGCACAG | 290 | 63 |
|  | R | CACGGTTCAGCCATCCCTGAG |  |  |
| *hlyA* | F | AACAAGGATAAGCACTGTTCTGGCT | 1177 | 62 |
|  | R | ACCATATAAGCGGTCATTCCCGTCA |  |  |
| *cnf-1* | F | AAGATGGAGTTTCCTATGCAGGAG | 498 | 56 |
|  | R | CATTCAGAGTCCTGCCCTCATTATT |  |  |
| Housekeeping gene for MLST alleles | | | | |
| *rpoB* | F | GGCGAAATGGCWGAGAACCA | 501 | 50 |
|  | R | GAGTCTTCGAAGTTGTAACC |  |  |
| *gapA* | F | TGAAATATGACTCCACTCACGG | 450 | 60 |
|  | R | CTTCAGAAGCGGCTTTGATGGCTT |  |  |
| *mdh* | F | CCCAACTCGCTTCAGGTTCA | 477 | 50 |
|  | R | CCGTTTTTCCCCAGCAGCAG |  |  |
| *pgi* | F | CTGCTGGCGCTGATCGGCAT | 432 | 50 |
|  | R | TTATAGCGGTTAATCAGGCCGT |  |  |
| *phoE* | F | ACCTACCGCAACACCGACTTCTTCGG | 420 | 50 |
|  | R | TGATCAGAACTGGTAGGTGAT |  |  |
| *infB* | F | ACTAAGGTTGCCTCCGGCGAAGC | 318 | 50 |
|  | R | CGCTTTCAGCTCAAGAACTTC |  |  |
| *tonB* | F | CTTTATACCTCGGTACATCAGGTT | 414 | 45 |
|  | R | ATTCGCCGGCTGRGCRGAGAG |  |  |
