# Supplementary material for: Dissemination and characteristics of carbapenem-resistant Klebsiella pneumoniae in nine district hospitals in southwestern China
Source: Front Microbiol. 2023 Oct 24;14:1269408. doi: 10.3389/fmicb.2023.1269408 (PMC10628634; doi:10.3389/fmicb.2023.1269408)
Supplement: Supplementary file 2 [file Table_2.doc]

**Supplementary Table 2** Clinical characteristics of 51 patients with CRKP infections

| **Bacterial strain** | **Gender/age(years)** | **Underlying conditions** | **Department** | **Inpatient days** | **Outcome** | **Infection acquired** |
| --- | --- | --- | --- | --- | --- | --- |
| CRKP-1 | M/84 | Sepsis | ICU | 28 | Survived | HCA |
| CRKP-2 | F/62 | Pulmonary infection | Res | 24 | Survived | HA |
| CRKP-3 | F/50 | Pulmonary infection; intracranial infection | Neu | 23 | Survived | HA |
| CRKP-4 | F/85 | Pulmonary infection; urinary tract infection; cerebral hemorrhage | Neu | 42 | Death | HA |
| CRKP-5 | F/67 | urinary tract infection | Nep | 20 | Survived | HCA |
| CRKP-6 | F/73 | Pulmonary infection; urinary tract infection | Res | 26 | Survived | HA |
| CRKP-7 | M/72 | Sepsis | Res | 30 | Survived | HCA |
| CRKP-8 | M/65 | Severe acute pancreatitis | Gas | 32 | Survived | HA |
| CRKP-9 | F/55 | Severe acute pancreatitis | Gas | 26 | Survived | HA |
| CRKP-10 | F/78 | Pulmonary infection | Res | 28 | Discontinued treatment | HCA |
| CRKP-11 | M/34 | Head injury | Cer | 31 | Survived | HA |
| CRKP-12 | F/78 | Sepsis | ICU | 46 | Discontinued treatment | HA |
| CRKP-13 | M/0.1 | Neonatal pneumonia | Neo | 18 | Survived | HA |
| CRKP-14 | M/67 | Severe pneumonia | Res | 32 | Survived | HCA |
| CRKP-15 | F/51 | Acute severe organophosphorus poisoning | Gas | 15 | Discontinued treatment | HA |
| CRKP-16 | F/58 | Severe pneumonia | Res | 27 | Survived | HA |
| CRKP-17 | F/63 | Head injury; subarachnoid hemorrhage | Cer | 33 | Survived | HA |
| CRKP-18 | M/71 | Pulmonary infection; Cerebral hemorrhage | Neu | 49 | Discontinued treatment | HA |
| CRKP-19 | M/90 | Pulmonary infection; urinary tract infection | Inf | 39 | Discontinued treatment | HCA |
| CRKP-20 | M/82 | Severe pneumonia; urinary tract infection | Res | 40 | Discontinued treatment | HCA |
| CRKP-21 | F/85 | Sepsis | Res | 51 | Discontinued treatment | HA |
| CRKP-22 | M/82 | Obstructive jaundice | Gas | 20 | Survived | HA |
| CRKP-23 | M/82 | Pulmonary infection | Res | 19 | Survived | HA |
| CRKP-24 | F/53 | Cancer of biliary duct | Hep | 62 | Death | HA |
| CRKP-25 | M/0.1 | Neonatal pneumonia | Neo | 19 | Survived | CA |
| CRKP-26 | F/62 | Urinary tract infection | Nep | 18 | Survived | HCA |
| CRKP-27 | F/81 | Severe pneumonia | Res | 54 | Death | HA |
| CRKP-28 | M/70 | Hyperplasia of prostate with acute urinary retention | Uri | 24 | Survived | HA |
| CRKP-29 | M/63 | Pulmonary infection; intracranial infection | Neu | 33 | Discontinued treatment | HA |
| CRKP-30 | M/79 | AECOPD | Res | 28 | Survived | HA |
| CRKP-31 | F/44 | Sepsis | ICU | 30 | Survived | HA |
| CRKP-32 | M/36 | Trauma | Tra | 25 | Survived | HA |
| CRKP-33 | M/70 | Pulmonary infection | Res | 28 | Survived | CA |
| CRKP-34 | M/43 | Head injury | Cer | 51 | Discontinued treatment | HA |
| CRKP-35 | M/45 | Multisite trauma | Tra | 54 | Discontinued treatment | HA |
| CRKP-36 | M/66 | Cerebral hemorrhage | Neu | 48 | Death | HA |
| CRKP-37 | M/54 | Head injury | Cer | 41 | Discontinued treatment | HA |
| CRKP-38 | M/68 | Cerebral hemorrhage | Neu | 44 | Discontinued treatment | HA |
| CRKP-39 | M/86 | Bronchopneumonia | Res | 27 | Survived | HA |
| CRKP-40 | M/67 | Pulmonary infection | Res | 24 | Survived | HA |
| CRKP-41 | M/45 | Head injury | Cer | 31 | Survived | HA |
| CRKP-42 | F/83 | Severe pneumonia | ICU | 52 | Death | HA |
| CRKP-43 | M/53 | Head injury | Cer | 34 | Survived | HA |
| CRKP-44 | M/81 | Lumbar disc herniation; Benign prostatic hyperplasia | Uri | 28 | Survived | HA |
| CRKP-45 | F/73 | Cerebral infarction; Respiratory failure | Neu | 57 | Death | HA |
| CRKP-46 | F/82 | Urinary tract infection | Nep | 25 | Survived | HA |
| CRKP-47 | M/76 | Severe acute pancreatitis | Gas | 42 | Discontinued treatment | HA |
| CRKP-48 | F/87 | Trauma | Tra | 25 | Survived | HA |
| CRKP-49 | M/47 | Pulmonary infection; renal failure | Nep | 40 | Survived | HA |
| CRKP-50 | M/79 | Pulmonary infection | Res | 27 | Survived | HA |
| CRKP-51 | F/56 | Urinary tract infection | Nep | 20 | Survived | CA |

Note: M, male; F, female; ECOPD, acute exacerbations of chronic obstructive pulmonary disease; Cer, cerebral surgery department; Gas, gastroenterology department; Hep, hepatological surgery department; Inf, infectious disease department; ICU, intensive care unit; Neo, neonatology department; Nep, nephrology department; Neu, neurology department; Res, respiratory medicine department; Tra, traumatology department; Uri, urinary surgery department；CA, community-acquired; HA, hospital-acquired; HCA, healthcare-associated.
